# Allergic tendencies are associated with larger gray matter volumes

**DOI:** 10.1038/s41598-018-21985-8

**Published:** 2018-02-27

**Authors:** Hikaru Takeuchi, Yasuyuki Taki, Rui Nouchi, Ryoichi Yokoyama, Yuka Kotozaki, Seishu Nakagawa, Atsushi Sekiguchi, Kunio Iizuka, Yuki Yamamoto, Sugiko Hanawa, Tsuyoshi Araki, Carlos Makoto Miyauchi, Kohei Sakaki, Takayuki Nozawa, Shigeyuki Ikeda, Susumu Yokota, Magistro Daniele, Yuko Sassa, Ryuta Kawashima

**Affiliations:** 1 Division of Developmental Cognitive Neuroscience, Institute of Development, Aging and Cancer, Tohoku University, Sendai, Japan; 2 Division of Medical Neuroimaging Analysis, Department of Community Medical Supports, Tohoku Medical Megabank Organization, Tohoku University, Sendai, Japan; 3 Department of Radiology and Nuclear Medicine, Institute of Development, Aging and Cancer, Tohoku University, Sendai, Japan; 4 Creative Interdisciplinary Research Division, Frontier Research Institute for Interdisciplinary Science, Tohoku University, Sendai, Japan; 5 Human and Social Response Research Division, International Research Institute of Disaster Science, Tohoku University, Sendai, Japan; 6 Department of Advanced Brain Science, Institute of Development, Aging and Cancer, Tohoku University, Sendai, Japan; 7 School of Medicine, Kobe University, Kobe, Japan; 8 Division of Clinical research, Medical-Industry Translational Research Center, Fukushima Medical University School of Medicine, Fukushima, Japan; 9 Department of Human Brain Science, Institute of Development, Aging and Cancer, Tohoku University, Sendai, Japan; 10 Department of Psychiatry, Tohoku Medical and Pharmaceutical University, Sendai, Japan; 11 Department of Psychosomatic Research, National Institute of Mental Health, National Center of Neurology and Psychiatry, Tokyo, Japan; 12 Department of Psychiatry, Tohoku University Graduate School of Medicine, Sendai, Japan; 13 ADVANTAGE Risk Management Co., Ltd., Sendai, Japan; 14 Graduate School of Arts and Sciences, Department of General Systems Studies, The University of Tokyo, Tokyo, Japan; 15 Department of Ubiquitous Sensing, Institute of Development, Aging and Cancer, Tohoku University, Sendai, Japan; 16 National Centre for Sport and Exercise Medicine (NCSEM), The NIHR Leicester-Loughborough Diet, Lifestyle and Physical Activity Biomedical Research Unit, School of Sport, Exercise, and Health Sciences, Loughborough University, Loughborough, England

## Abstract

Allergic tendencies are associated with important cognitive and physiological factors, such as intelligence and mathematical abilities. Allergies are widely prevalent, especially in modern life, and the reason for its association with important cognitive variables is an intriguing scientific question. However, despite the unique characteristics of cognitive correlates of allergy, the anatomical correlates of allergy remain unknown. The aim of this study was to identify the associations between regional gray matter volume (rGMV) and allergic tendencies in young adults. In a study cohort of 1,219 healthy, educated young adults, we identified a positive correlation between total allergic tendency and rGMV in large anatomical clusters that mainly encompassed the dorsal part of the cerebral neocortex, right anterior insula, and cerebellum. Furthermore,both mean rGMV of the entire part of these clusters and total allergenic tendency showed a significant positive correlation with spatial ability. These results suggest the link among allergic tendencies, larger rGMV, and the better spatial ability in healthy, educated young adults.

## Introduction

Allergic diseases are becoming increasingly common in the modern world and affect approximately 50% of the population in different forms^1^. The prevailing theory suggests that the underlying cause of allergies involves the facilitation of the immune response mediated by Th2immune cells^2^. Allergic tendencies (the degree to which one shows symptoms of certain types of allergies) are associated with a number of important physiological and cognitive factors. For example, previous studies showed a higher occurrence of allergies in intellectually or mathematically gifted boys^3,4^.

Further, the model of the traditional theory presented by McManus and Bryden^5^, which tried to explain the number of observations, included the indirect associations between (a) developmental changes in the brain, that are associated with increased spatial giftedness, and (b) retardation of growth of structures involved in immunity, which in turn leads to immune disorders, including various types of allergies.

However, despite the unique characteristics of cognitive correlates of allergy, and traditional theory linking spatial abilities and brain development^5^, the anatomical correlates of allergy remain unknown.

Although previous studies have not directly suggested the potential neural correlates of allergy, insights can be inferred from the wide range of studies conducted on other aforementioned observed or hypothetical correlates of allergy (such as mathematic competence, spatial ability, and intelligence). The analysis of regional cortical structures[regional gray matter volume (rGMV) and cortical thickness] is arguably the most widely used method to investigate the neural basis of individual differences in the aforementioned allergy correlates. For example, a previous study investigated whether individual math competence was associated with the regional gray matter volume of the left intraparietal sulcus^6^. In addition, the gray matter volume of several areas in the prefrontal and parietal areas in childhood shows positive correlations with math skills 6 years later^7^. Another study revealed that spatial cognitive ability, independent of general intelligence, is associated with larger rGMV in the parietal cortex^8^. In general, rGMV in the lateral areas of the frontal and parietal lobes are associated with intelligence, including spatial intelligence^9,10^. These areas have been suggested to play important roles in general higher order cognition and spatial and number cognition^11,12^.

Considering the aforementioned findings on higher intellectual and spatial abilities, we hypothesized that an increased rGMV in areas of the parietal cortices, precuneus, and, possibly, the prefrontal cortex would be associated with allergic tendency.

The purpose of this study was to test this hypothesis and reveal the association between rGMV and allergic tendency. For this purpose, we employed voxel-based morphometry (VBM)^13^. We also investigated the associations between allergic tendencies and various psychological variables to better understand the nature of allergy correlates.

## Methods

### Subjects

The present study, which is a part of an ongoing project to investigate the association between brain imaging, cognitive function, and aging, included 1219 healthy, right-handed individuals (703 men and 516 women) for whom allergy-related measures and structural data were collected. The mean age of the subjects was 20.7 years [standard deviation (SD) = 1.8; age range: 18–27 years]. The Supplemental Methods section includes the details of subjects’ information. The Supplemental Discussion includes the limitations of this study in relation to the subjects’ characteristics. The study protocols were performed in accordance with the Declaration of Helsinki (1991), and the study was approved by the Ethics Committee of the Tohoku University. All study participants provided their written informed consent.

### Allergy measurements

In this study, allergic tendencies were assessed through a self-reported questionnaire. The subjects were asked if they had each type of allergy through “yes” or “no” questions, and if they had the allergy, they were asked to describe the severity of the symptoms. In the latter question, there were 5 potential answers: (a) very severe, (b) relatively severe, (c) neither, (d) not so severe, and (e) not severe. If the subjects answer “no” to the first question, they do not have to answer the second question and the score is 0. However, if the subjects answer “yes” to the first question, they have to answer the second question. Answers (a), (b), (c), (d), and (e) correspond to scores 1, 2, 3, 4, and 5, respectively. The list of allergies included 7 types: (1) hay fever-allergic rhinitis, (2) hay fever-allergic conjunctivitis, (3) bronchial asthma, (4) atopic dermatitis, (5) egg allergy, (6) soy allergy, and (7) milk allergy. The questionnaire also included 4 fields for other unspecified allergies. Here subjects were asked to list other allergies that they had and their symptoms. Therefore the total number of items of allergy is 7 plus the number of items each subjects added for unspecified allergies.

Self-reported questionnaires for assessing allergies were utilized in two previous studies^4,14^, which are representative for our study theme; the rationale for our use of this questionnaire is described in the Supplemental Methods. The total allergy score, which is the summed score from all questionnaire items, was the main outcome measure used in our analysis. In this summation, the score of each question is equally added.

### Psychological measures

Neuropsychological tests were administered. The following detailed cognitive functions were also analyzed. These tests are described in this subsection and were largely reproduced from our previous studies^15–17^.

Measures of control (baseline) basic cognitive functions that are not shown to be associated with allergies, to the best of our knowledge, are as follows:

[A] Computerized digit span task is a working memory task, wherein the sum scores of computerized forward and backward digit span tests are calculated for assessing the working memory performance. As per the task, the subjects were asked to view a progressively increasing number of random digits that were visually presented at the rate of 1 digit/s on a computer screen. Next, they were asked to repeat the sequence by pressing number buttons on the screen in the presented order (digit span forward) or in the reverse order (digit span backward), starting from two digits. Three sequences were presented at each level until the participants responded incorrectly to all three sequences, at which point, the task was terminated. The score of each test was equal to the sum of the number of digits correctly repeated in the digit span forward and backward tasks.

[B] The perception factor of the Tanaka B-type intelligence test (TBIT)^18^ type 3B is a mass intelligence test employed for 3^rd^-year junior high school and older examinees. The perception speed factor of TBIT measures the simple processing speed and involves three subtests in which a subject solves as many simple problems as possible in a stipulated time frame (e.g., a few minutes). This factor involves three subtests: a displacement task [requiring substitution of a figure (nine figures) with a number (1–9) based on a model chart within 3 min], identification versus same–different judgment (Japanese kana characters; requiring judging of whether two meaningless Japanese strings are the same within 2 min), and marking figures [selecting types that are identical to three samples from a series (sequence) of eight different types within 2 min].

The measurement of cognitive functions that were hypothesized to be specifically associated with allergy are as follows:

The association of allergic tendency with psychometric measurement of general intelligence (determined by non-verbal reasoning) and with spatial ability was hypothesized based on known associations between intellectual giftedness and allergy^3,4^ as well as on the traditional hypothesis of the indirect association of allergy with the development of visuospatial abilities^5^.

Furthermore, the association of reading ability with allergic tendency was hypothesized based on the association between dyslexia and allergy^19,20^.

[C] Raven’s Advanced Progressive Matrices (RAPM)^21^ is a nonverbal reasoning task representative of the measure of general intelligence. Psychometric intelligence is considered to be relevant to allergies. RAPM (Raven, 1998) contains 36 nonverbal items that require fluid reasoning ability. Each item consists of a 3 × 3 matrix, with a missing piece that is completed by selecting the best of the 8 alternatives. The score of this test (derived from the number of correct answers given in 30 min) was used as an index of the individual psychometric measure of intelligence.

[D] Spatial relation factor of TBIT measures the spatial abilities to understand the relationship among different objects in space. In all subtests, the subjects are required to solve as many problems as possible within a stipulated time frame (for example, a few minutes). This factor involves three subtests: i) the maze test (requiring the tracing of a maze using a pencil from start to finish in 2 min), ii) the counting cubes (requiring the counting of cubes piled up in a three-dimensional manner within 2 min), and iii) filling in figures (requiring completion of incomplete figures in comparison with the sample figures when rotated within 3 min).

[E] Reasoning factor of TBIT measures reasoning based on the filling of the numbers in a sequence (requiring the filling of the blanks of a number sequence with suitable numbers as per the rules of the number arrangement task within 3 min).

[F] Reading comprehension task was developed by Kondo *et al*.^22^ and involves 8 sections of articles, with each article having 4 questions and each question having 5 choices of answers. The questions were designed to allow the subjects to determine the correct answers after reading the articles correctly. The subjects were asked to correctly answer as many questions as possible in 13 min. Similar to the reading comprehension task developed in Western countries, the score obtained in this task has a significant positive correlation with that obtained in the reading span task. The details of this test, including information on its development and validity, are available elsewhere^22^.

The data on control cognitive functions were added for reference and comparisons because if only the data on spatial function are provided, we are unable to determine whether the association of allergy with spatial functions is specific to this cognitive function.

### Psychological data analysis

The psychological data were analyzed by SPSS 18.0 statistical software (SPSS Inc., Chicago, IL). The sex difference in demographic variables was determined by two-tailed *t*-tests. For each analysis, *P* < 0.05 was considered to be statistically significant. Because these were only descriptive analyses irrelevant to the objective of this study, no corrections were performed for multiple comparisons. The associations between total allergy scores and psychological measures were analyzed using multiple regression analyses, with age and sex as covariates. In these analyses, the results with a threshold of *P* < 0.05 were considered to be statistically significant after correcting for the false discovery rate (FDR) using the graphically sharpened method^23^. Standardized partial regression coefficients were reported for the effect size.

### Image acquisition

The methods for MR image acquisition have been described in our previous study^24^. In brief, all MRI data acquisition was performed using a 3-T Philips Achieva Scanner. High-resolution T1-weighted structural images (T1WIs:240 × 240 matrix, TR = 6.5 ms, TE = 3 ms, FOV = 24 cm, slices = 162, slice thickness = 1.0 mm) were collected using a magnetization-prepared rapid gradient echo sequence.

### Pre-processing of structural data

The structural data were preprocessed using Statistical Parametric Mapping software (SPM12; Wellcome Department of Cognitive Neurology, London, UK) implemented in MatLab (Mathworks Inc., Natick, MA, USA). We used the new segmentation algorithm and diffeomorphic anatomical registration through exponentiated Lie algebra (DARTEL) registration process implemented in SPM12. The T1-weighted structural images of each individual were segmented and normalized to the Montreal Neurological Institute (MNI) space for generating images with 1.5 × 1.5 × 1.5 mm^3^ voxels. Moreover, a volume change correction (modulation)^25^ was performed. Subsequently, the generated rGMV images were smoothed by convolving them with an isotropic Gaussian kernel of 8-mm full width at half maximum (FWHM). The full description of these procedures is available in the Supplemental Methods.

### Whole-brain statistical analysis

The association of rGMV with individual differences in the overall allergic tendency was assessed. The statistical analysis of the imaging data was performed with SPM8. In these analyses, a whole-brain multiple regression analysis was performed. The analyses was performed with sex, age, and total intracranial volume (TIV) calculated as described previously^26^ as confounding variables. The total allergy scores were used as covariates, and only voxels with an rGMV signal intensity of >0.05 were used for all participants.

A multiple comparison correction was performed by threshold-free cluster enhancement (TFCE)^27^ with randomized (5,000 permutations) nonparametric testing using the TFCE toolbox (http://dbm.neuro.uni-jena.de/tfce/). A threshold of FWE corrected at *P* < 0.05 was used.

SPM8 was used here because of the better compatibility of the software of TFCE and the homemade script for analyses. As long as TFCE is used, the rationale for estimating the statistical significance is the same, and this would not matter to the results.

A study of voxel-wise analyses for correlations between the spatial cognitive function and rGMV was published elsewhere^28^.

### Post-hoc correlation analyses for assessing the variables in this study

Next, we assessed the associations among each allergic tendency, the total allergy score, significant rGMV correlates of the total allergy score in the abovementioned whole brain analysis, and significant psychological correlates of the total allergy score to reveal the relationship among these.

The correlations among these were assessed using simple correlation analyses, and a correlation matrix table was constructed.

For significant rGMV correlates of the total allergy score in the abovementioned whole brain analysis, the mean rGMV values of the entire part ofsignificant clusters (all voxels of significant correlations) in the abovementioned whole brain analysis were extracted using log-roi-batch v2.0 (http://www.aimfeld.ch/).

## Results

### Descriptive statistics

The mean and standard deviation for age, general intelligence test score, total allergy score, and individual allergy scores are presented in Table 1. The distribution of the total allergy score for men and women in the present study are presented in Table 2.

**Table 1:** The demographics of study participants.

Measure	Male (N = 703)		Female (N = 516)	
	Mean	SD	Mean	SD
Age	20.80	1.89	20.60	1.60
RAPM	28.79	3.86	28.10	3.81
Allergy total*	2.93	3.20	2.87	3.59
Hay fever-allergic rhinitis	1.58	1.66	1.35	1.63
Hay fever-allergic conjunctivitis	0.59	1.24	0.68	1.26
Asthma	0.15	0.49	0.11	0.44
Atopic dermatitis	0.3	0.81	0.34	0.86
Egg allergy	0.02	0.22	0.03	0.25
Soy allergy	0.00	0.04	0.00	0.00
Milk allergy	0.01	0.15	0.02	0.17

*Sum of the allergy scores of the allergies listed in the table as well as other self-reported allergies.

**Table 2:** Distribution of total allergic tendency in men and women.

	0	1–2	3–4	5–6	7–8	9–10	11–12	13–14	15–16	17–18	19+
Male	247	134	156	66	62	21	9	3	2	3	0
Female	216	82	84	60	36	23	6	5	2	0	2

### Whole-brain analyses of the correlations between overall allergic tendency and rGMV

**Figure 1:**
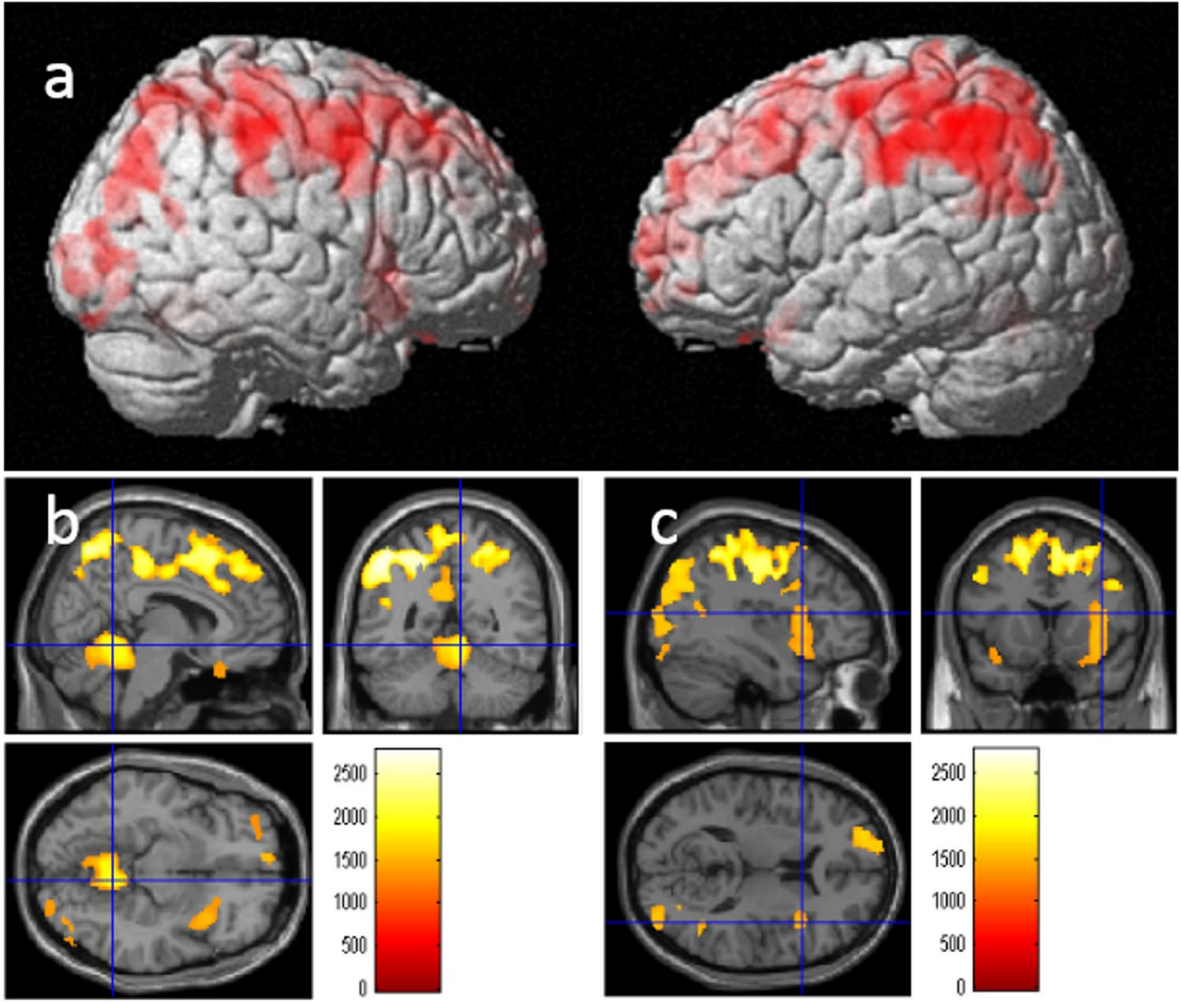
The positive rGMV correlates of overall allergic tendency. (**a**,**b**, and **c**) The results were obtained using a threshold of threshold-free cluster enhancement (TFCE) of *P* < 0.05 based on 5,000 permutations. The results were corrected at the whole-brain level. (**a**) Regions with significant correlations are projected on a rendered SPM8 image. Significant positive correlations with rGMV were observed in extensive cortical areas, especially in the dorsal part of the cerebral neocortex. (**b**) Regions with significant correlations are overlaid on a “single subject” T1 image from SPM8. Significant positive correlations with rGMV were observed across the widespread areas. Color bar represents TFCE score.

A whole-brain multiple regression analysis showed that the total allergic tendency significantly and positively correlated with rGMV in widespread areas involving several clusters. Areas of correlation mainly included the dorsal part of the cerebral neocortex, right anterior insula, and cerebellum (Fig. 1, Table 3, Supplemental Table 1). The same analysis showed there were no significant negative correlations between the allergy tendency and rGMV. Sex differences in the anatomical correlates of allergy were not the main purpose of the study; however, our supplemental investigation of this parameter yielded no significant results (Supplemental Methods and Results).

**Table 3:** Brain regions that exhibited a significant positive correlation between total allergic tendency and rGMV.

	Included gray matter areas*(number of significant voxels in left and right side of each anatomical area)	x	y	z	TFCE value	Corrected *p* value (FWE)	Cluster size (voxel)
1	Angular gyrus (L:1186, R:915)/Calcarine Cortex (R:254)/Anterior cingulum (L:238, R:82)/Middle cingulum (L:1803, R:2011)/Posterior cingulum (L:376, R:24)/Cuneus (L:27, R:120)/Inferior frontal operculum (L:74, R:319)/Inferior frontal triangular (L:6)/Middle frontal medial area (L:209)/Middle frontal other areas (L:2968, R:1877)/Superior frontal medial area (L:1737, R:997)/Superior frontal orbital area (L:125)/Superior frontal other areas (L:3724, R:1448)/Fusiform gyrus (R:10)/Lingual gyrus (R:109)/Inferior occipital lobe (R:589)/Middle occipital lobe (L:409, R:2031)/Superior occipital lobe (R:298)/Paracentral lobule (L:1056, R:357)/Inferior parietal lobule (L:4156, R:1075)/Superior parietal lobule (L:1546, R:1540)/Postcentral gyrus (L:3695, R:2229)/Precentral gyrus (L:2613, R:3594)/Precuneus (L:3870, R:1716)/Rectus gyrus (L:4)/Supplemental motor area (L:1845, R:1818)/Supramarginal gyrus (L:713, R:1385)/Middle temporal gyrus (L:323, R:304)/Superior temporal gyrus (R:1)/	−42	−36	57	2769.96	0.002	62788
2	Lingual gyrus (L:3, R:30)/Thalamus (L:1185, R:1094)/	7.5	−49.5	−10.5	1978.17	0.011	4252
3	Amygdala (R:6)/Inferior frontal operculum (R:136)/Inferior frontal orbital area (R:170)/Inferior frontal triangular (R:1)/Superior frontal orbital area (R:1)/Insula (R:1087)/Parahippocampal gyrus (R:31)/Putamen (R:76)/Temporal pole (R:36)/	39	13.5	12	1546.18	0.025	2056
4	Inferior frontal orbital area (L:17)/Insula (L:16)/Temporal pole (L:216)/	−36	18	−24	1372.64	0.039	271
5	Superior frontal orbital area (L:2, R:6)/Rectus gyrus (L:63, R:107)/	6	27	−27	1363.41	0.039	480
6	Inferior frontal operculum (L:1)/	18	−43.5	58.5	1327.97	0.044	6
7	Inferior frontal orbital area (L:4)/Middle frontal orbital area (L:166)/Superior frontal orbital area (L:7)/	−30	49.5	−7.5	1310.14	0.047	169
8	Superior parietal lobule (R:1)/Postcentral gyrus (R:4)/	−37.5	0	22.5	1286.30	0.050	4

*Labelings of the anatomical regions of gray matter were based on the WFU PickAtlas Tool (http://www.fmri.wfubmc.edu/cms/software#PickAtlas/)^43,44^ and on the PickAtlas automated anatomical labeling atlas option^45^. Temporal pole areas included all subregions in the areas of this atlas.

### Psychological analyses of the correlations between overall allergic tendency and cognitive function

The total allergy tendency significantly and positively correlated with spatial ability (spatial relation factor of the TBIT) (β (standardized partial regression coefficient) = 0.082; P = 0.005, uncorrected; and P = 0.035, corrected for multiple comparisons using FDR), but not the other cognitive functions that were investigated. The total allergy tendency positively correlated with the psychometric intelligence test that consists of subtasks in which the subjects have to solve as many simple problems as possible within a certain time frame (e.g., a few minutes) at the uncorrected level (total score of the TBIT) but not the corrected level (β = 0.065; P = 0.029, uncorrected; and P = 0.102, corrected). There were no significant correlations between total allergic tendency and nonverbal reasoning ability (Raven’s advance progressive matrix test), numerical reasoning (reasoning factor of the TBIT), reading comprehension ability (reading comprehension test), simple processing speed (perceptual factor of the TBIT), or working memory (digit span). All statistical results are presented in Table 4. Sex differences in the correlates of allergy were not the main purpose of the study; however, our supplemental investigation of this parameter yielded no significant results (Supplemental Methods and Results).

**Table 4:** Statistical results (beta value (standardized partial regression coefficient), *t*-value, uncorrected *p*-values, *p*-value corrected for FDR^a^) for the multiple regression analyses performed using psychological variables and the covariates of age, sex, and allergic tendency as dependent variables.

Dependent variables	N	β	*t*	Allergic tendency	*p (FDR)*
				*p (uncorrected)*	
Digit span	1213	0.017	0.593	0.553	0.806
Perception speed factor of TBIT^b^	1095	0.054	1.774	0.076	0.177
Total intelligence score of TBIT	1095	0.065	2.185	0.029	0.102
Spatial relation factor of TBIT	1101	0.082	2.788	0.005	0.035
Reasoning factor of TBIT	1101	−0.006	−0.195	0.845	0.845
RAPM^c^	1219	−0.015	−0.512	0.608	0.806
Reading comprehension	1034	0.012	0.398	0.691	0.806

^a^False discovery rate. ^b^Tanaka B-type intelligence test, ^c^Raven’s advanced progressive matrices (a general intelligence task).

### Correlations among total allergy score, individual allergic tendencies, the mean rGMV of the entire part of significant clusters in the whole-brain analyses, and spatial function

We also assessed the associations among each allergic tendency, the total allergy score, the mean rGMV of the whole part of aforementioned significant anatomical clusters (all voxels of significant correlations), and spatial cognition. The results indicated that hay fever-allergic rhinitis showed the strongest correlation with total allergic tendency, and only this allergy correlated with spatial function and mean rGMV of the entire part of significant clusters in the whole-brain analyses (Supplemental Table 2).

## Discussion

In this study, the relationships between rGMV, psychological measures, and allergic tendencies were investigated. Partly consistent with our hypothesis, our novel findings showed that overall allergic tendencies were associated with a greater rGMV in extensive cortical areas, especially in the dorsal part of the cerebral neocortex, right anterior insula, and cerebellum. Furthermore, allergic tendencies showed a positive correlation with spatial ability.

Our findings showed that dorsal part of the cerebral neocortex was associated with overall allergic tendency, and we speculate that this may be related to the increased spatial ability in subjects with allergies. The posterior part of the inferior parietal region is important for spatial attention and spatial function^29^, and the posterior parietal cortex represents the mental representation of the visual world^30^. The PFC can regulate the focusing of attention to different parts of space^29^ and is suggested to dissociate and manipulate visuospatial information^31^. The posterior part of the inferior parietal region and the PFC are connected through the superior longitudinal fasciculus^29^, and they may work together during visuospatial functions. Through these regions, increased allergic tendencies may be associated with the increased visuospatial ability observed in subjects with increased allergies. Partly consistent with this notion, one of our recent studies^28^ showed that the spatial factor positively correlated with allergic tendencies in widespread brain areas when the total intracranial volume was not regressed out, including areas where rGMV and allergy tendency were significantly correlated. A similar finding was observed in another study that showed a positive correlation between spatial intelligence and rGMV of widespread brain areas,including dorsal frontal and parietal areas that showed significant correlation between rGMV and allergy tendency^10^. In addition, another previous study showed that the visuospatial cognitive ability that is independent of general intelligence, is positively correlated with parietal volume^8^.

It is difficult to conclude larger rGMV is better in young adults. rGMV is just a volume and like greater body volume does not always mean healthy or adaptive state, multiple mechanisms (lack of adaptive developmental pruning, adaptive increase in neural tissues such as glia, or less damage or degeneration of neural tissues) can underline greater rGMV. While greater rGMV is associated with greater brain size, which, in turn, is associated with greater intelligence^32^ (greater cognitive competence may be associated with greater rGMV in this case), during the adolescence development is associated with advanced cortical thinning and superior intelligence is shown to be associated with greater cortical thinning(greater cognitive competence may beassociated with reduced rGMV in this aspect)^33^. Previously, using a data of a huge sample, we have shown greater general intelligence is associated with larger rGMV in widespread areas^28^ (greater cognitive competence may be associated with greater rGMV in this case), but on the other hand, smaller rGMV was shown to be associated with greater empathizing^34^ (greater socioemotional competence is likely to be associated with less rGMV in this case). These data suggest that it is difficult to assume greater rGMV or that less rGMV reflects cognitive or neural competence from rGMV alone. Here we interpreted allergy tendency’s association with greater rGMV is associated with better functioning of the relevant regions based on the data that allergic tendency is associated with greater spatial ability.

We cannot determine the causal relationship or micro-level mechanisms of the observed associations between rGMV and allergies from the cross-sectional macro-level neuroimaging studies. However, for reference, we provide one possible speculative mechanism. A traditional model to explain the empirical link among certain cognitive abilities, allergy, and other variables assumed that fetal testosterone plays a key role in forming associations among variables^5^. In this model, fetal testosterone leads to the development of the brain, which is associated with greater spatial and mathematical ability, and the retardation of the immune system, which leads to immune disorders including allergy^5^. Thus, this model may explain the present findings of the associations among allergy, spatial ability, and rGMV. It is a hypothetical model, and though famous, it has received criticism^35^. Further, the model assumes that fetal testosterone leads to increased growth of the posterior side of the right hemisphere of the brain and delayed growth of the posterior side of the left hemisphere of the brain, both of which are assumed to lead to greater spatial ability^5^. However, a previous study has shown that fetal testosterone exposure level is positively correlated with rGMV of the bilateral lateral prefrontal cortex and parietal areas^36^, which is partly incongruent with the abovementioned traditional model. Consistent with the abovementioned model are recent studies that have provided evidence that the fetal testosterone level is associated with some visuospatial abilities^37–39^. Considering these, one speculative mechanism to explain the observed associations among rGMV, allergy, and spatial ability is that (a) a higher fetal testosterone level is associated with greater rGMV in the frontal and parietal areas, spatial abilities, and allergic tendencies and (b) this, in turn, forms the associations among greater rGMV in the frontal and parietal areas, greater spatial abilities, and increased allergic tendencies. In this case, even if the association of allergy with rGMV is related to association of allergy with spatial ability, it does not necessarily mean that allergy is good for the brain. The associations could be caused by a fourth factor (fetal testosterone level in this hypothetical model) affecting allergic tendency, spatial abilities, and rGMV. On the other hand, the immune aspect of the model, which assumed that fetal testosterone exposure leads to retardation of the immune system and leads to allergy and atopic diseases, has been criticized as it is not congruent with consequent physiological findings^40^. Therefore, to the best of our knowledge, to date, the exact mechanisms that can clearly explain the present findings well are not clear. Our present study did not gather data of fetal testosterone exposure because of limited research resources. Future studies need to be conducted to reveal this mechanism.

Our findings showed that somatosensory areas (postcentral gyrus and anterior insula) were associated with overall allergic tendency, and we speculate that this may be relevant to the differences in somatosensory neural mechanisms observed in subjects with allergic tendencies. The postcentral gyrus is involved in the processing of sensory information, and the anterior insula and ACC are involved in the affective processing of pain and disgust^41^. Furthermore, the rGMV of these areas is related to pain disorders^41^. In addition, although the cerebellum has multiple functions, including somatosensory and pain-related functions^42^, it is suggested to be involved in emotional aspects of pain, anticipation of pain, the inhibition of nocifensive behaviors, and others^42^. Thus, one can speculate that sensitivity in these processes may lead to allergies or that sensory experiences related to allergies may lead to an increase in the gray matter volume of these areas. However, we did not gather these measures in our current cross-sectional study. Thus, we are unable to confirm these speculations.

In conclusion, greater allergic tendencies were associated with a greater rGMV in areas related to higher order cognition and improved spatial abilities. A substantial number of people in the modern world suffer from allergies. These results suggest the link among allergic tendencies, larger rGMV, and the better spatial ability in healthy, educated young adults. Future studies are needed to determine the mechanisms underlying the observed associations.

## Electronic supplementary material

Supplementary material
