# Supplementary material for: Allergic tendencies are associated with larger gray matter volumes
Source: Sci Rep. 2018 Feb 27;8:3694. doi: 10.1038/s41598-018-21985-8 (PMC5829247; doi:10.1038/s41598-018-21985-8)
Supplement: Supplementary file 1 — Supplementary material [file 41598_2018_21985_MOESM1_ESM.pdf]

Supplemental online material

Allergic tendencies are associated with larger gray matter volumes

Hikaru Takeuchi[a], Yasuyuki Taki[a,b,c], Rui Nouchi[d,e,f], Ryoichi Yokoyama[g], Yuka Kotozaki[h], Seishu Nakagawa[i,j], Atsushi Sekiguchi[b,i,k], Kunio Iizuka[l], Yuki Yamamoto[i], Sugiko Hanawa[i], Tsuyoshi Araki[m], Carlos Makoto Miyauchi[n], Kohei Sakaki[f], Takayuki Nozawa[o], Shigeyuki Ikeda[o], Susumu Yokota[a], Magistro Daniele[p], Yuko Sassa[a], Ryuta Kawashima[a,f,i]

[a]*Division of Developmental Cognitive Neuroscience, Institute of Development, Aging and Cancer, Tohoku University, Sendai, Japan*

[b]*Division of Medical Neuroimaging Analysis, Department of Community Medical Supports, Tohoku Medical Megabank Organization, Tohoku University, Sendai, Japan*

[c]*Department of Radiology and Nuclear Medicine, Institute of Development, Aging and Cancer, Tohoku University, Sendai, Japan*

[d]*Creative Interdisciplinary Research Division, Frontier Research Institute for Interdisciplinary Science, Tohoku University, Sendai, Japan*

[e]*Human and Social Response Research Division, International Research Institute of Disaster Science, Tohoku University, Sendai, Japan*

[f]*Department of Advanced Brain Science, Institute of Development, Aging and Cancer, Tohoku University, Sendai, Japan*

[g] *School of Medicine, Kobe University, Kobe, Japan*

[h]*Division of Clinical research, Medical-Industry Translational Research Center, Fukushima Medical University School of Medicine, Fukushima, Japan*

[i]*Department of Human Brain Science, Institute of Development, Aging and Cancer, Tohoku University, Sendai, Japan*

[j]*Department of Psychiatry, Tohoku Medical and Pharmaceutical University, Sendai,*

Japan

[k]*Department of Adult Mental Health, National Institute of Mental Health, National Center of Neurology and Psychiatry, Tokyo, Japan*

[l]*Department of Psychiatry, Tohoku University Graduate School of Medicine, Sendai, Japan*

[m]*ADVANTAGE Risk Management Co., Ltd.*

[n]*Graduate School of Arts and Sciences, Department of General Systems Studies, The University of Tokyo, Tokyo, Japan*

[o]*Department of Ubiquitous Sensing, Institute of Development, Aging and Cancer, Tohoku University, Sendai, Japan*

[p]*National Centre for Sport and Exercise Medicine (NCSEM), The NIHR Leicester-Loughborough Diet, Lifestyle and Physical Activity Biomedical Research Unit, School of Sport, Exercise, and Health Sciences, Loughborough University, England*

Corresponding author:

Hikaru Takeuchi

Division of Developmental Cognitive Neuroscience, IDAC, Tohoku University

4-1 Seiryo-cho, Aoba-ku, Sendai 980-8575, Japan

Tel/Fax: +81-22-717-7988

E-mail: takehi@idac.tohoku.ac.jp

Short title: Allergy, spatial function, and gray matter

Keywords: allergy, gray matter, spatial function, frontal lobe, asthma, hay fever

Supplemental Methods

Subjects. The present study, which is a part of an ongoing project to investigate the association between brain imaging, cognitive function, and aging, included 1219 healthy, right-handed individuals (703 men and 516 women) for whom relevant allergy-related measures and structural imaging data were collected. The mean (± standard deviation, SD) age of the subjects was 20.7 ± 1.8 years (age range, 18–27 years). The following descriptions were mostly reproduced from another study of ours from the same project using the exactly same methods regarding these issues [1]. Psychological tests and MRI scans not described in this study were performed together with those described in this study. All subjects were university students, postgraduates, or university graduates of less than one year's standing. All subjects had normal vision and none had a history of neurological or psychiatric illness.

Handedness was evaluated using the Edinburgh Handedness Inventory [2]. We can say we used a score > 0 as the cutoff value. However, because the right-handed subjects were recruited through recruit informations of the bulletin board for the job and the email, almost all the left-handed subjects were removed in that process. Regardless, the previous VBM study with a large sample size failed to find any handedness effects on the regional morphology [3]; thus, the effects of handedness on the regional morphology remain elusive.

Written informed consent was obtained from each subject. For nonadult subjects, written informed consent was obtained from their parents (guardians). This study was approved by the Ethics Committee of Tohoku University.

Subjects were instructed to get sufficient sleep, maintain their conditions, eat sufficient breakfast, and to consume their normal amounts of caffeinated foods and

drinks in the day of cognitive tests and MRI scans. In addition, subjects were instructed to avoid alcohol the night before the assessment.

Rationale for the use of self-reported measures to assess allergic tendencies

When assessing allergies, it is difficult to obtain doctors' diagnoses because it is unlikely that people who suffer from common allergies, such as hay fever, go to the hospital. Furthermore, to our knowledge, there are no established psychological measures to assess allergies in Japan.

Like other researches involving the association between allergy, asthma and cognition, the vast majority of relevant studies (the associations between hay fever, allergy, and cancer etc) have been performed with self-reported questionnaires [4]. Another study [5] developed and assessed a different type of questionnaire to measure allergic rhinitis. Using a specialist's diagnosis of allergic rhinitis together with a positive skin prick test, they found 74% sensitivity and 83% specificity for their questionnaire. Our questionnaire is different; however, we have no reason to believe that the difference between the present questionnaire and a positive skin prick test affected the significant findings in such a way that they form false significant associations.

Pre-processing of structural data. Preprocessing of the structural data was performed using Statistical Parametric Mapping software (SPM12; Wellcome Department of Cognitive Neurology, London, UK) implemented in Matlab (Mathworks Inc., Natick, MA, USA). Using the new segmentation algorithm implemented in SPM12, T1-weighted structural images of each individual were segmented into 6 tissues. In this new

segmentation process, default parameters were used, except that the Thorough Clean option was used to eliminate any odd voxel, affine regularization was performed with the International Consortium for Brain Mapping template for East Asian brains, and the sampling distance was set at 1 mm. We then proceeded to the diffeomorphic anatomical registration through exponentiated lie algebra (DARTEL) registration process implemented in SPM12. We used DARTEL import images of the 2 TPMs from the abovementioned new segmentation process. First, the template for the DARTEL procedures was created using imaging data from 800 participants (400 males and 400 females). The following methods were the same as in our previous study and descriptions were reproduced from our previous study [6] (These subjects were the first 400 male and 400 female subjects in this project). Next, using this existing template, the DARTEL procedures were performed for all of the subjects in the present study. In these procedures, default parameter settings were used. The resulting images were spatially normalized to the Montreal Neurological Institute (MNI) space to give images with $1.5 \times 1.5 \times 1.5$ mm^3 voxels. In addition, we performed a volume change correction (modulation) by modulating each voxel with the Jacobian determinants derived from spatial normalization, which allowed us to determine regional differences in the absolute amount of brain tissue [7]. Subsequently, all images were smoothed by convolving them with an isotropic Gaussian kernel of 8 mm full width at half maximum (FWHM).

The effects of an interaction between sex and allergic tendency on cognitive function and rGMV

To investigate if the psychological and anatomical correlates of allergic tendency

differ between sexes, we performed an analysis of covariance (ANCOVA). Psychological analyses were performed in PASW Statistics 22 (SPSS Inc.). The dependent variables in these analyses were same as those used in the multiple regression analysis for investigation of the correlation with total allergic tendency across sexes. In the ANCOVA, sex was a fixed factor, and age, overall allergic tendency, and the interaction between sex and overall allergic tendency were added to the model.

To investigate the interaction effects of sex and individual overall allergic tendencies in a whole-brain analysis, we used a voxel-wise analysis of covariance (ANCOVA) in which sex difference was a group factor (using the full factorial option in SPM8). In this analysis, age, total intracranial volume, and overall allergic tendency were covariates, and age and overall allergic tendency were modeled to enable unique relationships with rGMV (using the interactions option in SPM8) for each sex. Total intracranial volume was modeled so that it has a common relationship with rGMV across sexes. The interaction effects between sex and overall allergic tendency were assessed using t-contrasts. A correction for multiple comparisons was performed with the same method used for the whole-brain multiple regression analysis.

Supplemental Results

The effects of an interaction between sex and allergic tendency on cognitive function and rGMV

There were no significant effects of interaction between sex and allergic tendency in the psychological or whole-brain analyses.

Supplemental Discussion

Limitations of this study

The present study had some limitations. Subjects in this study were young and healthy and consisted of mostly undergraduate or postgraduate students. This kind of limited sampling is a common hazard for studies using college students [8]. Thus, whether the present findings can be applied to the full population should be determined in future studies. However, for the purpose of this study, focusing on subjects with relatively high psychometric intelligence was important. This is because testosterone levels were previously described as relevant to allergic tendencies, and poor and high cognitive functioning are associated with higher testosterone levels [9-11]. Thus, studying a more educated, and perhaps more intelligent [12], group was necessary for a simple and sensitive analysis. However, the average RAPM score, which was measured using a time limit of 30 min, was >28. This score is much >18, which was the score for the 50 percentile of a normative sample of Poland's 19 year olds published in 1991 [13] [notably, the normative sample's general intelligence increased as time passed (Flynn effect) [13]]. The higher psychometric intelligence in our present group may weaken the association between allergic tendency and psychometric intelligence observed in previous studies. In addition, we did not measure circulating or prenatal testosterone levels. This was because of the limited research resources from multiple perspectives, and omission of this measurement did not hinder our fulfillment of the study's purpose. However, determining the association between testosterone levels and the variables used in the study, which were described in previous studies and outlined in the Introduction section, could enrich our study.

Supplemental table 1.

Anatomical classifications of the peaks and subpeaks of brain regions that exhibited a significant positive correlation between total allergic tendency and rGMV

	Anatomical classification[a]	x	y	z	TFCE value	Corrected p value (FWE)	Cluster size (voxel)
cluster 1	Left Postcentral gyrus	-42	-36	57	2769.957	0.0016	62788
	Left inferior parietal lobule	-48	-48	46.5	2654.722	0.002	
	Left Inferior parietal lobule	-46.5	-51	49.5	2648.363	0.002	
	Left Inferior parietal lobule	-51	-43.5	49.5	2632.026	0.002	
	Left Inferior parietal lobule	-43.5	-49.5	54	2624.337	0.002	
	NA	-21	-46.5	49.5	2562.941	0.002	
	Left Inferior parietal lobule	-25.5	-46.5	51	2560.338	0.002	
	Left Inferior parietal lobule	-30	-45	51	2560.338	0.002	
	Left Inferior parietal lobule	-30	-52.5	51	2551.763	0.002	

Left Precuneus	-4.5	-64.5	61.5	2537.269	0.002
Right Superior parietal lobule	16.5	-66	58.5	2505.312	0.0024
Right Superior parietal lobule	13.5	-60	57	2489.336	0.0024
Left Superior parietal lobule	-16.5	-64.5	61.5	2460.843	0.0026
Left Superior parietal lobule	-21	-61.5	61.5	2450.186	0.0026
Right Precuneus	4.5	-64.5	55.5	2439.724	0.0028
NA	-19.5	-45	43.5	2438.653	0.0028
NA	-19.5	-51	45	2437.297	0.0028
Right Superior parietal lobule	28.5	-61.5	61.5	2408.321	0.0028
Left Supramarginal gyrus	-58.5	-24	42	2387.508	0.0032
Left Postcentral gyrus	-36	-40.5	66	2386.6	0.0032
Left Precuneus	-10.5	-66	49.5	2374.591	0.0032
Left Precuneus	-10.5	-67.5	54	2372.277	0.0032
Left Inferior parietal lobule	-43.5	-36	40.5	2369.675	0.0032
Left Postcentral gyrus	-48	-33	45	2366.587	0.0032

	Right Middle frontal other areas	27	28.5	46.5	2336.849	0.0038	
	Right Supplemental motor area	6	19.5	52.5	2320.917	0.0038	
	Right Superior frontal other areas	16.5	30	34.5	2317.427	0.0038	
	Right Superior frontal other areas	16.5	12	49.5	2302.388	0.0044	
	NA	-60	-42	42	2300.896	0.0044	
	Left Angular gyrus	-42	-64.5	46.5	2300.33	0.0044	
	Right Superior frontal other areas	16.5	22.5	39	2300.166	0.0044	
	Right Superior frontal other areas	16.5	21	45	2296.092	0.0046	
cluster 2	Right Thalamus	7.5	-49.5	-10.5	1978.167	0.011	4252
	Vermis	-3	-55.5	-10.5	1871.597	0.0142	
	Right Thalamus	9	-63	-15	1603.92	0.0234	
	Vermis	6	-66	-16.5	1582.001	0.0242	
	Vermis	1.5	-67.5	-16.5	1559.814	0.0246	
	Left Thalamus	-6	-66	-10.5	1446.588	0.0322	
	NA	-7.5	-64.5	-28.5	1379.706	0.0386	

	Vermis	-3	-66	-27	1379.706	0.0386	
	NA	10.5	-64.5	-27	1355.977	0.0404	
	Left Thalamus	-4.5	-75	-15	1333.444	0.0432	
clulster 3	Right Inferior frontal operculum	39	13.5	12	1546.177	0.0252	2056
	Right Insula	36	16.5	-15	1541.962	0.0258	
	Right Insula	39	13.5	0	1527.153	0.0262	
	Right olfactory bulb	27	9	-21	1481.739	0.0296	
	Right Insula	31.5	12	-19.5	1480.567	0.0296	
	NA	22.5	24	-10.5	1356.547	0.0404	
	Right Inferior frontal orbital area	28.5	22.5	-15	1353.75	0.0404	
cluster 4	Left Temporal pole	-36	18	-24	1372.636	0.039	271
	Left Temporal pole	-37.5	15	-19.5	1371.128	0.039	
cluster 5	Right Rectus gyrus	6	27	-27	1363.406	0.0394	480
	NA	-10.5	25.5	-33	1339.005	0.0424	

	Left Rectus gyrus	-3	31.5	-25.5	1327.964	0.044	
cluster 6	Right Superior parietal lobule	18	-43.5	58.5	1327.967	0.044	6
	Right Postcentral gyrus	18	-43.5	63	1327.967	0.044	
cluster 7	Left Middle frontal orbital area	-30	49.5	-7.5	1310.144	0.0468	169
	Left Middle frontal orbital area	-25.5	54	-10.5	1302.107	0.0478	
cluster 8	Left Inferior frontal operculum	-37.5	0	22.5	1286.303	0.0496	4

*Labelings of the anatomical regions of gray matter were based on the WFU PickAtlas Tool (http://www.fmri.wfubmc.edu/cms/software#PickAtlas/) [14, 15] and on the PickAtlas automated anatomical labeling atlas option [16]. Temporal pole areas included all subregions in the areas of this atlas.

Supplemental Table 2. Simple correlation coefficients and p-values for the associations between each allergy score and the significant allergy correlates in this study.

	1	2	3	4	5	6	7	8	9	10
1 Allergy total	-		*	**	***					
2 Hay fever-allergic rhinitis	0.790, $9.17*10^{-261}$***	-								
3 Hay fever-allergic conjunctivitis	0.724, $2.16*10^{-198}$***	0.589, $9.26*10^{-115}$***	-							
4 Bronchial asthma	0.304, $1.91*10^{-27}$***	0.119, $3.00*10^{-5}$***	0.052, 0.071	-						
5 Atopic dermatitis	0.377, $1.80*10^{-42}$***	0.072, 0.012*	0.037, 0.193	0.193, $1.11*10^{-11}$***	-					
6 Egg allergy	0.206, $3.70*10^{-13}$***	0.015, 0.593	0.009, 0.751	0.051, 0.076	0.195, $6.66*10^{-12}$***	-				
7 Soy allergy	0.094,	−0.026,	−0.014,	−0.008,	0.126,	0.496,	-			

	0.001**	0.370	0.614	0.780	$9.81*10^{-6}$***	$7.87*10^{-77}$***				
8 Milk allergy	0.114,	0.009,	0.005,	0.030,	0.096,	0.417,	−0.003,	-		
	$6.30*10^{-5}$***	0.760	0.854	0.288	0.001**	$1.34*10^{-52}$***	0.930			
9 Mean rGMV of all	0.105,	0.115,	0.044,	0.047,	0.029,	0.001,	0.001,	0.013,	-	
significant clusters	$2.51*10^{-4}$***	$5.79*10^{-5}$ ***	0.123	0.099	0.315	0.095	0.094	0.651		
10 Spatial factor of	0.087,	0.072,	−0.025,	0.001,	0.035,	0.030,	0.042,	0.035,	0.161,	-
TBIT	0.004**	0.017*	0.410	0.098	0.248	0.065	0.160	0.242	$8.09*10^{-8}$***	

*p < 0.05, ** p < 0.01, *** p < 0.001

References

1. Takeuchi, H. et al. Degree centrality and fractional amplitude of low-frequency oscillations associated with Stroop interference. *Neuroimage* **119**, 197-209 (2015).
2. Oldfield, R.C. The assessment and analysis of handedness: the Edinburgh inventory. *Neuropsychologia* **9**, 97-113 (1971).
3. Good, C.D. et al. Cerebral asymmetry and the effects of sex and handedness on brain structure: a voxel-based morphometric analysis of 465 normal adult human brains. *Neuroimage* **14**, 685-700 (2001).
4. Turner, M.C. et al. Cancer mortality among US men and women with asthma and hay fever. *Am. J. Epidemiol.* **162**, 212-221 (2005).
5. Annesi‐Maesano, I. et al. The score for allergic rhinitis (SFAR): a simple and valid assessment method in population studies. *Allergy* **57**, 107-114 (2002).
6. Takeuchi, H. et al. The structure of the amygdala associates with human sexual permissiveness: Evidence from voxel-based morphometry. *Hum. Brain Mapp.* **36**, 440-448 (2015).
7. Ashburner, J. & Friston, K.J. Voxel-based morphometry-the methods. *Neuroimage* **11**, 805-821 (2000).
8. Jung, R.E. et al. Neuroanatomy of creativity. *Hum. Brain Mapp.* **31**, 398-409 (2010).
9. Ostatníková, D., Putz, Z., Celec, P. & Hodosy, J. May testosterone levels and their fluctuations influence cognitive performance in humans. *Scr. Med. (Brno).* **75**, 245-254 (2002).
10. Ostatníková, D. et al. Intelligence and salivary testosterone levels in prepubertal children. *Neuropsychologia* **45**, 1378-1385 (2007).

11. Gouchie, C. & Kimura, D. The relationship between testosterone levels and cognitive ability patterns. *Psychoneuroendocrinology* **16**, 323-334 (1991).

12. Tambs, K., Sundet, J.M., Magnus, P. & Berg, K. Genetic and environmental contributions to the covariance between occupational status, educational attainment, and IQ: A study of twins. *Behav. Genet.* **19**, 209-222 (1989).

13. Raven, J. Manual for Raven's progressive matrices and vocabulary scales. (Oxford Psychologists Press, Oxford, 1998).

14. Maldjian, J.A., Laurienti, P.J. & Burdette, J.H. Precentral gyrus discrepancy in electronic versions of the Talairach atlas. *Neuroimage* **21**, 450-455 (2004).

15. Maldjian, J.A., Laurienti, P.J., Kraft, R.A. & Burdette, J.H. An automated method for neuroanatomic and cytoarchitectonic atlas-based interrogation of fMRI data sets. *Neuroimage* **19**, 1233-1239 (2003).

16. Tzourio-Mazoyer, N. et al. Automated anatomical labeling of activations in SPM using a macroscopic anatomical parcellation of the MNI MRI single-subject brain. *Neuroimage* **15**, 273-289 (2002).
